# Data in support of poisoning related mortalities from southern Himachal Pradesh

**DOI:** 10.1016/j.dib.2017.04.028

**Published:** 2017-04-29

**Authors:** Shivkant Sharma, Kuldeep Kumar, Saurabh Bhargava, V.S. Jamwal, Arun Sharma, Rajvinder Singh

**Affiliations:** a Department of Genetics, M.D. University, Rohtak 124001, Haryana, India; b State Forensic Science Laboratory, Junga 171218, Himachal Pradesh, India

**Keywords:** Poisoning, Himachal Pradesh, Shimla, Solan, Sirmaur, Kinnaur, Database

## Abstract

Poisoning has always been pointed as one of the leading causes of human death throughout the world. Despite the best efforts made by many research institutes, the worldwide true figure on mortalities with poisoning could never be achieved due to many reasons. One of the main reasons is the unavailability of complete database from the rural and catchment areas of the world where these types of incidents are usual. People can be made aware about this problem by presenting data articles on regular basis, therefore to mark a resource document these data should be regularly up-dated. The current data report is a briefing of types and trends of chemical poisoning amongst human in southern hilly region of Himachal Pradesh (HP), India. This research database is an outcome of five year retrospective study based on assessment of records pertaining human deaths associated with poisoning occurred in southern Himachal Pradesh, and reported at State Forensic Science Laboratory (SFSL), Junga during 2010-14. Cases where ethyl alcohol was detected have been put under exclusion criterion. All the cases were reviewed and summarized in terms of yearly and monthly frequency of reports wrapping important information portraying the involvement of gender, age, locality, types of poison, and mode of death in the poisoning incidents. Review of these scientific reports showed some notable figures having a direct concern with public and legal domains to promote risk reduction and prevention of chemical poisonings.

**Specifications Table**

**Table:** 

Subject area	Forensic Science
More specific subject area	Toxicology
Type of data	Figure and Tables
How data was acquired	Surveyed at SFSL, Junga (HP) with kind permission of authority.
Data format	Filtered and analyzed
Experimental factors	Nil
Experimental features	Experimental design included tabulation of frequency in number and percentile of occurrence of factors based on different epidemiological parameters.
Data source location	Himachal Pradesh (30° 22.40 N to 33° 12.40 N latitude and 75° 45.55E to 79° 04.20E longitude).
Data accessibility	Data are available with this article.

**Value of the data**

• Routine monitoring of deaths due to poisoning is an essential exercise to refreshing the database from all corners of the world. So, the current data become of high value as not much literature is available on chemical poisoning in human from HP.
• The data provided here is first of its kind information for readers to fully understand types and trends of poisoning prevailing in this part of the world.
• The information contained in this data report is intended for general use to assist public knowledge and research as well. These data are also a source of information for the state and native poison control centres and many other institutes to conduct research and strategize to deal this type of problem.

## 1 Data

**Fig. 1:**
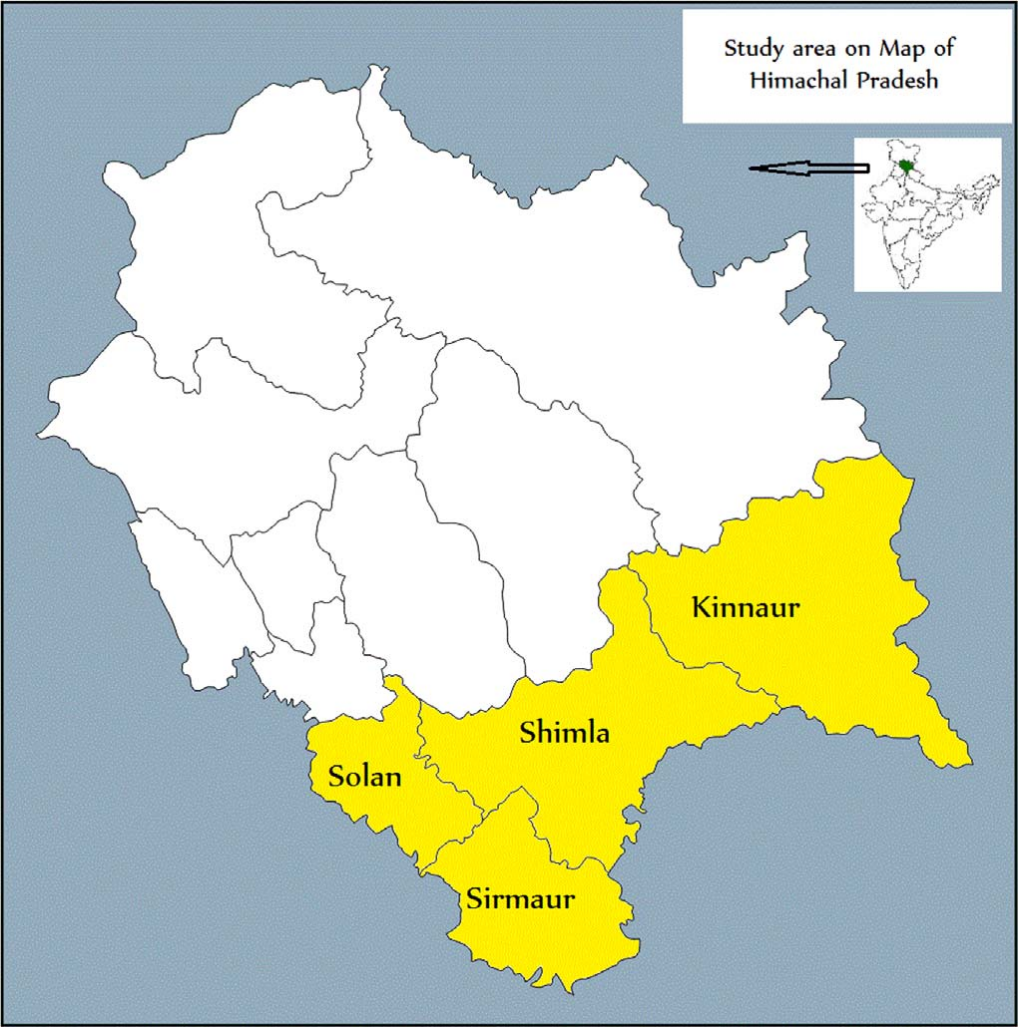
Map of Himachal Pradesh showing study area.

Due to easy availability, the use of chemical poisons has remained one of the most common ways of ending human lives by any mode of death. Large numbers of people die every year due to poisoning, especially acute pesticide poisoning [1]. A very high number of fatalities also occur in India due to poisoning [2], [3], [4], [5], [6]. A concise piece of information on poisoning is available from HP [7], [8], [9], [10], [11]. Therefore, the data provided in this article explored some significant inferences on mortalities associated with poisoning from southern HP. This data is limited only to those cases which were registered by the law enforcement agencies from Shimla, Solan, Sirmaur and Kinnaur districts situated in the southern region of HP (Fig. 1). All related particulars were obtained from First Information Report (FIR), Post-mortem Report (PMR), and Toxicology reports.

## 2 Experimental design, materials and methods

The mountainous state of Himachal Pradesh is situated in the western Himalayan region of India. It comprises an area of 55,673 square kilometres divided into 12 districts which are further grouped into three divisions namely Shimla, Kangra and Mandi. The division of Shimla controls Shimla, Kinnaur, Sirmaur and Solan districts located in the southern region of this state. This division is a habitat of ~29% of total population and same coverage of geographical area of the state. These four districts come under the jurisdiction of SFSL established in Junga town. Post-mortem samples including blood, urine, viscera, gastric lavage or vomits material of victim obtained while autopsy are sent to this laboratory for chemical analysis of poisonous substances (if present any). The present database is inference of cases reported at SFSL, Junga from 1st January, 2010 up to 31st December, 2014. Data analysis involved all kinds of chemical poisoning.

Database revealed 1291 positive reports out of 2721 total cases submitted during study period. Data presented herein is a realistic information depicting year wise reporting of positive cases from all selected districts (Table 1), gender (male & female) and locality (urban & rural) wise difference (Table 2), age groups affected along with gender difference (Table 3), types of poisons involved in different districts (Table 4), district wise poisons involved in different age groups (Table 5), and gender wise modes of death (Table 6). Data revealed that ethyl alcohol was utterly detected in 669 (~52%) cases from all districts during 2010–14. It is important to note that ethyl alcohol was vastly detected in the bodies met with road accidents but it was irresolute to establish death due to alcohol impairment in actual. Use of pesticide including *Dichlorovos* and *Paraquat* was reported in majority (~30%) of cases. Out of 18 cases of paraquat poisoning, the maximum (10) cases were reported in year 2013 from all districts. Use of aluminum phosphide or zinc phosphide was reported in 12 cases, whereas in rest of the cases only phosphine was mentioned as poisonous substance.

## Figures and Tables

**Table 1:** Cases reported year wise from selected districts during 2010–2014.

**Year**	**Total cases reported**	**Positive Cases**	**Cases reported from districts**				**Percentage**
			**Shimla**	**Solan**	**Sirmaur**	**Kinnaur**	**out of 1291**
2010	566	256	114	58	50	34	19.83%
2011	521	256	107	86	39	24	19.83%
2012	481	235	115	65	38	17	18.20%
2013	521	258	105	90	44	19	19.98%
2014	632	286	117	97	48	24	22.15%
Total	2721	1291	558	396	219	118	
**Percentage**			**43.22%**	**30.67%**	**16.96%**	**9.14%**	**100%**

**Table 2:** Gender and locality wise difference.

	**Shimla**		**Solan**		**Sirmaur**		**Kinnaur**		**Total**		**Total**
									**(%)**		**(%)**
	Male	Female	Male	Female	Male	Female	Male	Female	Male	Female	
**Rural**	293	80	198	61	104	27	58	12	653	180	833
									(78.4%)	(21.6%)	(64.5%)
**Urban**	176	9	127	10	68	20	37	11	408	50	458
									(89.1%)	(10.9%)	(35.5%)
**Total**	469	89	325	71	172	47	95	23	1061	230	1291
**%**	84.05%	15.95%	82.07%	17.93%	78.5%	21.5%	80.5%	19.5%	82.18%	17.82%	100%

**Table 3:** Age group along with gender involved.

**Age Group**	**Shimla**	**Solan**	**Sirmaur**	**Kinnaur**	**Gender**		**Total**
					Male	Female	
Child (0–14 years)	10	08	02	04	13 (54.16%)	11 (45.84%)	24 (1.85%)
Young Adult	122	100	60	29	206 (66.24%)	105 (33.76%)	311 (24.1%)
(15–25 years)							
Adult	238	160	100	54	475 (86.05%)	77 (13.95%)	552 (42.75%)
(26–40 years)							
Middle Age	160	114	47	25	318 (91.90%)	28 (8.10%)	346 (26.8%)
(41–59 years)							
Old Age	28	14	10	06	49 (84.48%)	9 (15.52%)	58 (4.5%)
(60 years and above)							
Total	558	396	219	118	1061	230	1291

**Table 4:** District wise number of cases and types of poisons involved.

**Type of poison used**	**Shimla**	**Solan**	**Sirmaur**	**Kinnaur**	**Total**	**%**
Ethyl Alcohol	322	203	87	57	669	51.82
Insecticide/Pesticides (excluding Phosphine)	170	102	73	45	390	30.20
Phosphine	27	46	39	07	119	9.21
Ethyl Alcohol + Insecticide/Pesticides	18	26	11	06	61	4.72
Ethyl Alcohol + Phosphine Gas	07	07	01	00	15	1.61
Prescription Drugs	02	04	04	00	10	0.77
aOthers	12	08	04	03	27	2.09
Total	558	396	219	118	1291	100%

a **others:** volatile solvents (kerosene, toluene and paraffin), inorganic acids, carbon-monoxide, copper sulphate, mercuric chloride, atropine, and blends of ethyl alcohol with carbon monoxide/prescription drugs

**Table 5:** Types of poisons involved in different age groups.

**Age Group**	**Ethyl alcohol**	**Pesticide/insecticide**	**Phosphine**	**Ethyl alcohol and Pesticide including phosphine**	**Others**	**Total**
Child (0–14 years)	01	15	06	00	02	24
Young Adult (15–25 years)	105	149	39	08	10	311
Adult (26–40 years)	317	147	39	36	13	552
Middle Age (41–59 years)	218	66	31	27	04	346
Old Age (60 and above)	30	19	07	02	00	58
Total	671	396	122	73	29	1291

**Table 6:** Modes of death and gender involved.

**Mode of Death**	**Male**	**Female**	**Total**
Suicidal	414 (65.50)	218 (34.50)	632
Accidental	593 (98.34)	10 (1.66)	603
Homicidal	54 (84.37)	02 (15.63)	56
Total	1061	230	1291
